# Visualization of complex DNA double-strand breaks in a tumor treated with carbon ion radiotherapy

**DOI:** 10.1038/srep22275

**Published:** 2016-03-01

**Authors:** Takahiro Oike, Atsuko Niimi, Noriyuki Okonogi, Kazutoshi Murata, Akihiko Matsumura, Shin-Ei Noda, Daijiro Kobayashi, Mototaro Iwanaga, Keisuke Tsuchida, Tatsuaki Kanai, Tatsuya Ohno, Atsushi Shibata, Takashi Nakano

**Affiliations:** 1 Department of Radiation Oncology, Gunma University Graduate School of Medicine, 3-39-22, Showa-machi, Maebashi, Gunma, 371-8511, Japan; 2 Gunma University Heavy Ion Medical Center, 3-39-22, Showa-machi, Maebashi, Gunma, 371-8511, Japan; 3 Research Program for Heavy Ion Therapy, Division of Integrated Oncology Research, Gunma University Initiative for Advanced Research, 3-39-22, Showa-machi, Maebashi, Gunma, 371-8511, Japan; 4 Advanced Scientific Research Leaders Development Unit, Gunma University, 3-39-22, Showa-machi, Maebashi, Gunma, 371-8511, Japan

## Abstract

Carbon ion radiotherapy shows great potential as a cure for X-ray-resistant tumors. Basic research suggests that the strong cell-killing effect induced by carbon ions is based on their ability to cause complex DNA double-strand breaks (DSBs). However, evidence supporting the formation of complex DSBs in actual patients is lacking. Here, we used advanced high-resolution microscopy with deconvolution to show that complex DSBs are formed in a human tumor clinically treated with carbon ion radiotherapy, but not in a tumor treated with X-ray radiotherapy. Furthermore, analysis using a physics model suggested that the complexity of radiotherapy-induced DSBs is related to linear energy transfer, which is much higher for carbon ion beams than for X-rays. Visualization of complex DSBs in clinical specimens will help us to understand the anti-tumor effects of carbon ion radiotherapy.

Radiotherapy is an essential tool for cancer treatment. Over 50% of all patients with localized malignant tumors receive radiotherapy as part of their initial treatment, either alone or in combination with surgery and chemotherapy. Carbon ion radiotherapy is an emerging technique in radiotherapy and is a potential breakthrough in the field. Carbon ions show a sharp dose distribution, called a Bragg peak, which enables the radiation to be highly concentrated within a tumor while at the same time sparing the surrounding normal tissues 1. Carbon ions at the Bragg peak show high linear energy transfer (LET), thereby providing a high density of energy deposition per unit length^2^. This results in a cell-killing effect that is 2–3 times greater than that of X-rays^3^. Clinical trials show that carbon ion radiotherapy has excellent anti-tumor effects against many cancer types, even tumors considered to be resistant to conventional X-ray radiotherapy, such as pancreatic cancer and osteosarcoma^4^,^5^. Thus far, more than 10000 patients have received carbon ion radiotherapy in eight facilities worldwide, and the number of the facilities under construction (or being considered for construction) is increasing, including in the United States^6^.

DNA double-strand breaks (DSBs) are major cytotoxic lesions induced by ionizing radiation. Basic research using cell-based *in vitro* experimental systems or physics models suggests that heavy ions with high LET, such as carbon ions, induce highly complex DNA lesions comprising DSBs, single strand breaks (SSBs), and base damage within 20–30 bp regions measuring 10–20 nm^7^,^8^. A recent study using advanced high-resolution microscopic technology visualized the formation of multiple close-proximity DSBs (hereafter referred to as “complex DSBs”) as clusters of γH2AX foci after high LET irradiation^9^. These clustered foci are a hallmark of high LET radiation. Compared with the single DSBs induced by X-rays, complex DSBs are hardly ever repaired. These irreparable complex DSBs are likely to lead to cell death^9^,^10^,^11^. Therefore, induction of complex DSBs may be the rationale underlying the greater anti-tumor effects of carbon ion radiotherapy when compared with X-ray radiotherapy. However, so far, no reports have provided evidence supporting the formation of complex DSBs in human tumors treated with clinical carbon ion radiotherapy. Moreover, the quality of DSBs in clinical tumors has not been analyzed using high-resolution imaging technology, not even those induced by X-ray radiotherapy (although several studies demonstrate the formation of DSBs in tumors treated with X-ray radiotherapy)^12^,^13^,^14^,^15^. Here, we aimed to visualize the formation of complex DSBs in a tumor treated with carbon ion radiotherapy, which can only be achieved using fresh biopsy specimens and advanced high-resolution microscopic technology.

## Results and discussion

**Figure 1:**
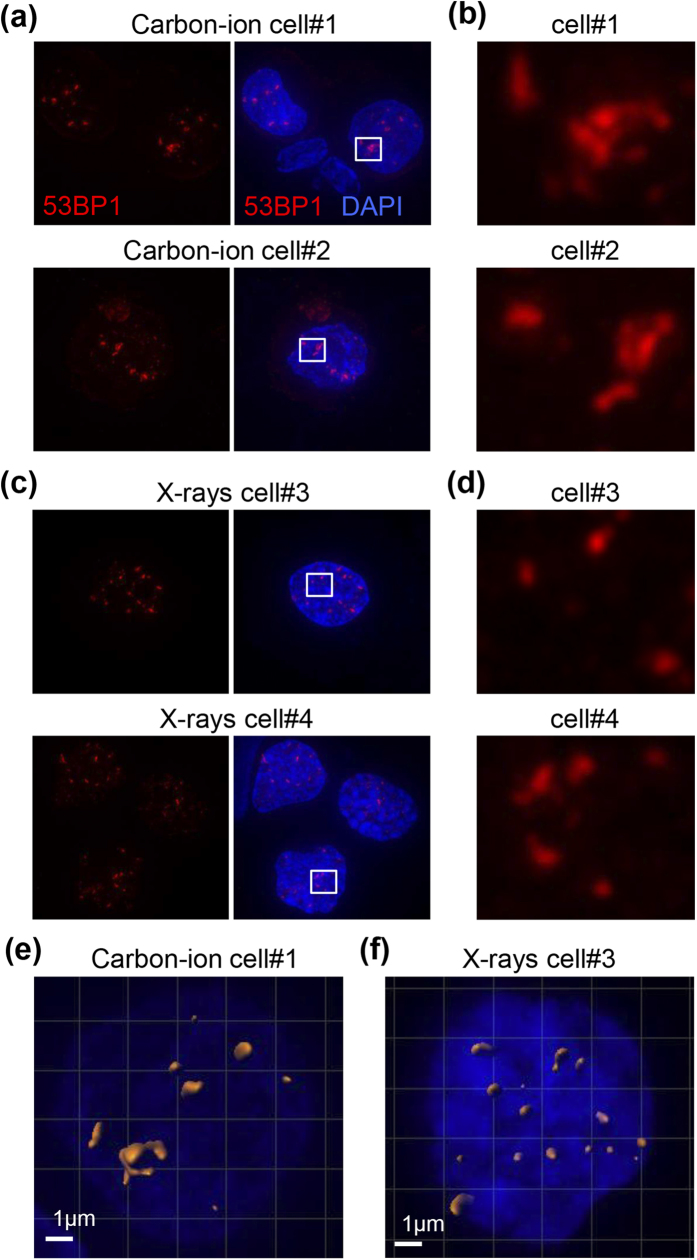
53BP1 foci induced by clinical carbon ion or X-ray radiotherapy in cancer cells. Tumor tissues were obtained from the uterine cervix by punch biopsy 30 min after the initial dose of irradiation. Tissues were stained with 53BP1 and DAPI and visualized using an advanced high-resolution microscopy technique with deconvolution. (**a**) Representative images of 53BP1 foci in a carbon ion-irradiated specimen. (**b**) Enlarged image of the area indicated by the white box in (**a**). (**c**) Representative images of 53BP1 foci in an X-ray-irradiated specimen. (**d**) Enlarged image of the area indicated by the white box in (**c**). (**e**,**f**) Polygon images of a carbon ion- (**e**) and X-ray- (**f**) irradiated cell. The 3D polygon image of 53BP1 foci was created using Imaris 8.0.1.

To visualize DSBs induced by clinical carbon ion and X-ray radiotherapy, we performed immunofluorescent staining of tumor biopsy specimens taken 30 min after the first radiation exposure with an antibody against p53-binding protein 1 (53BP1); foci formed by 53BP1 are a specific marker for DSBs (see Supplementary Discussion for a detailed description of the analytical method). Patient characteristics and clinical information are presented in Supplementary Table 1 and Supplementary Figure 1. 53BP1 foci were rarely observed in non-irradiated specimens (Supplementary Fig. 2). Notably, advanced high-resolution microscopic imaging with deconvolution revealed diffuse and clustered 53BP1 foci in the carbon ion-irradiated specimen (Fig. 1a,b). This morphology is consistent with the clustering of γH2AX foci, another DSB marker, after high LET irradiation *in vitro*^9^. By contrast, clustered 53BP1 foci were rarely observed in the X-ray-irradiated specimens (Fig. 1c,d). Three dimensional polygon images clearly demonstrated the formation of clustered foci in the carbon ion-irradiated specimen, but more uniform distribution of small foci in the X-ray-irradiated specimen (Fig. 1e,f and Supplementary Movie 1, 2).

**Figure 2:**
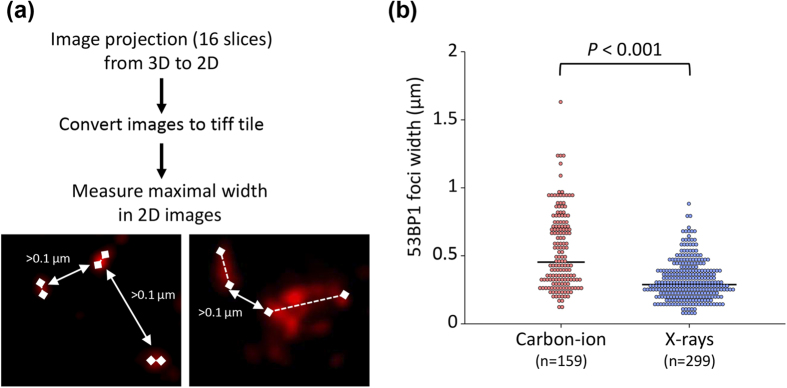
Size of 53BP1 foci induced by clinical carbon ion or X-ray radiotherapy in cancer cells. (**a**) Method used to measure the size of 53BP1 foci. Following image capture of 16 slices along the z-axis, all images were stacked into a single-layer with deconvolution. As shown in the panels, foci were defined as “individual” when they were separated from adjacent foci by >1 μm (left panel, X-rays; right panel, carbon ions). Solid lines represent the distance between foci; dashed lines represent maximum foci width. The maximum width of the 53BP1 foci in the z-stack images was measured using NIH ImageJ 1.48 v. (**b**) The distribution of the 53BP1 foci according to width is shown in the dot plots. The significance of the differences in the diameter of 53BP1 foci after carbon ion and X-ray radiotherapy were tested using the Mann–Whitney U test. Black bars indicate median values.

Next, to statistically analyze differences in the size of 53BP1 foci induced by carbon ion radiotherapy and those induced by X-ray radiotherapy, we measured the maximal width of the 53BP1 foci (Fig. 2a). In agreement with the morphological observations, we found that the maximal width of the 53BP1 foci in the carbon ion-irradiated specimen was significantly greater than that in the X-ray-irradiated specimen (Fig. 2b). Taken together, the results of imaging analyses support the formation of clustered 53BP1 foci, which represent the complex DSBs that are a hallmark of high LET radiation, in the patient treated with carbon ion radiotherapy.

**Figure 3:**
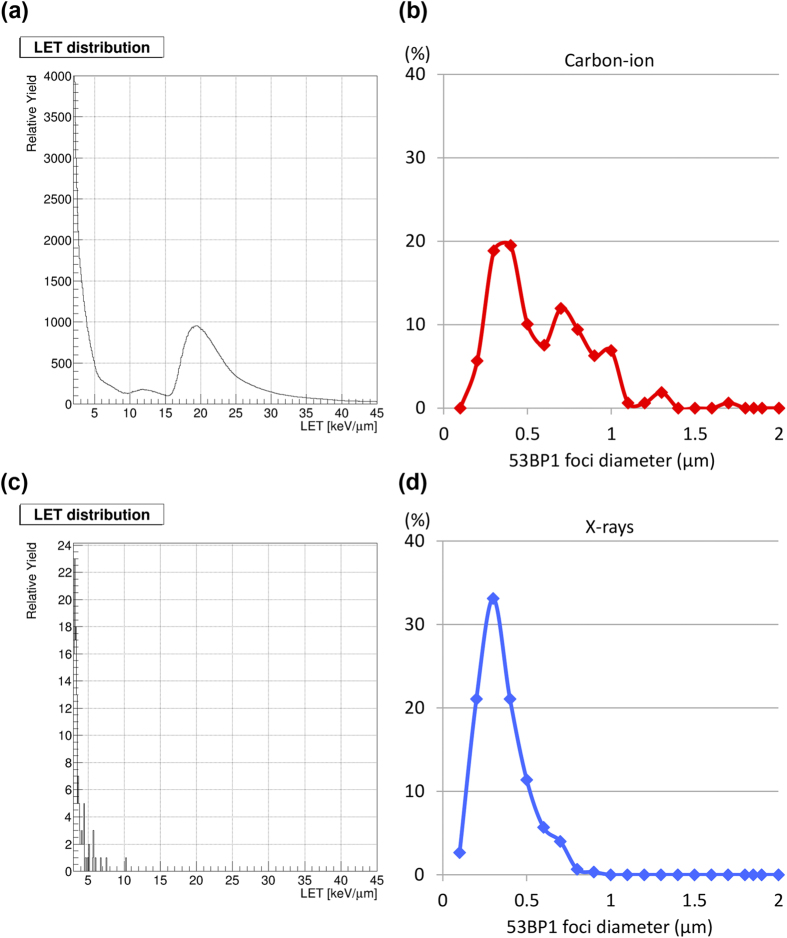
Distribution of LET and 53BP1 foci size after carbon ion or X-ray radiotherapy. (**a**,**c**) LET distribution of carbon ion beams at the center of the SOBP (**a**) or of 10 MV X-rays at a depth of 96 mm (**c**), calculated using a physical simulation method (see the Methods section for details). (**b**,**d**) The distribution and number of 53BP1 foci according to 53BP1 width in carbon ion- (**b**) or X-ray-irradiated (**d**) specimens. Note that the data for the 53BP1 foci are the same as those in Fig. 2, but in a different context.

Although we identified diffuse and clustered 53BP1 foci in the carbon ion-irradiated specimens, not all of the 53BP1 foci were clustered; a subset of 53BP1 foci was as small as those observed in X-ray-irradiated specimens (Figs 1 and 2). Technically, clinical carbon ion beams are delivered using either a passive modulation method or an active spot-scanning method^1^. In the passive modulation method (used by ourselves and several other facilities), the spread-out Bragg peak (SOBP), adjusted to the tumor size, is generated by integrating mono-energetic carbon ion beams that shift gradually in depth (Supplementary Fig. 3). The LET in a mono-energetic beam changes markedly according to depth, with a sharp peak around the Bragg peak (Supplementary Fig. 4). Hence, at a given point in the SOBP, cancer cells receive a mixture of beams with varying LET. Thus, we speculated that the variation in 53BP1 foci size may be correlated with LET distribution, i.e., different levels of DSB complexity may be caused by the wide distribution of LET in the tumor. To examine this issue, we calculated the LET distribution in the SOBP by physical simulation; for simplicity, we calculated the LET distribution at the center of the SOBP (see the Methods section for the details of the physical simulation method). The LET distribution at the center of the SOBP showed bimodal peaks (Fig. 3a). The peak with the higher LET corresponded to the carbon ion beams themselves, whereas that with the lower LET was attributable to secondary charged particles generated by the nuclear reaction (Supplementary Fig. 5). Surprisingly, the 53BP1 foci size distribution also showed bimodal peaks (Fig. 3b) that resembled the LET distribution. Furthermore, the size distribution of the 53BP1 foci in the X-ray-irradiated specimen also resembled the physically simulated LET distribution of X-rays (Fig. 3c,d). These data indicate that the complexity of DSBs induced by carbon ion and X-ray radiotherapy is related to the LET of the beams, supporting the concept that high LET carbon ion irradiation induces complex DSBs not only in cell-based experimental systems but also in a clinical setting.

To the best of our knowledge, this is the first study to successfully visualize DSBs induced by clinical carbon ion radiotherapy in a human tumor. The diffuse and clustered 53BP1 foci observed in the carbon ion-irradiated specimen, the size of which was greater than that of the X-ray-irradiated specimen, suggested that complex DSBs are induced by carbon ion radiotherapy. Pre-clinical research demonstrates that the repair efficacy of complex DSBs induced by high LET heavy ions is low^9^,^10^,^11^,^16^,^17^, and that the cell-killing effect of heavy ions is LET-dependent^18^,^19^. These data indicate that complex DSBs contribute to the greater anti-tumor effect of carbon ion radiotherapy compared with X-ray radiotherapy. Further research on a larger number of samples is required to confirm the results of this pilot study.

The distribution of SOBP carbon ion LET and the size of 53BP1 foci both showed bimodal peaks. This indicates that a subset of DSBs that form smaller 53BP1 foci were generated by low LET secondary charged particles during carbon ion therapy (Fig. 3, Supplementary Fig. 5). Interestingly, analysis of the distribution of the size of 53BP1 foci revealed that the lower peak in the carbon ion-irradiated specimen was consistent with the peak in the X-ray-irradiated specimen (Supplementary Fig. 6). Importantly, X-ray-induced non-complex DSBs are efficiently repaired by intrinsic DSB repair machinery; for example, >90% of DSBs induced by 3 Gy X-rays are repaired within 24 h post-irradiation^20^. This indicates that DSBs induced by the secondary charged particles generated during carbon ion irradiation with the passive modulation method are unlikely to contribute to the cell-killing effect of SOBP carbon ion irradiation, which is 2–3 times greater than that of X-rays^10^,^11^. From this point of view, our data point to the possibility that the active-scanning method, which generates fewer secondary charged particles than the passive modulation method, yields more “*bona fide* lethal” complex DSBs at an equivalent dose.

The beam prescription for radiotherapy treatments using X-rays and charged particles is based on the radiation dose. This led to the development of a treatment concept called “intensity-modulated radiation therapy”, in which the radiation dose is modulated such that it concentrates within a tumor and spares as much of the surrounding normal tissues as possible^21^. The data presented herein indicate that the complexity of DSBs induced by carbon ion irradiation is LET-dependent not only in a pre-clinical experimental setting but also in a clinical setting. Notably, the LET distribution within clinical carbon ion beams varies according to the depth of penetration into a tumor. In SOBP carbon ion beams generated by the passive modulation method, the proportion of high LET beams is greater at the distal end than at the proximal end^22^ (Supplementary Fig. 4, 7). Thus, treatments that utilize multiple SOBP carbon ion beams from multiple directions likely focus a lower proportion of high LET beams at the center of a tumor than at the tumor edge. Since pre-clinical research has identified an association between DSB complexity and cell-killing effect, this may result in a lower anti-tumor effect at the center of a tumor, particularly when treating bulky tumors. LET heterogeneity may also occur when using the active spot-scanning method as long as the treatment plan is normalized according to dose. These data raise the novel concept of “LET-intensity-modulated carbon ion radiotherapy”. For example, for SOBP carbon ion irradiation, addition of an “irradiation boost” to the center of the tumor using high LET spot-scanning beams that induce complex DSBs may increase local control of the tumor, while at the same time exposing the surrounding normal tissues to less toxic low LET radiation. Further research using animal xenograft models will be necessary to examine the association between the LET of carbon ion beams and the complexity and lethality of DSBs induced by them.

It should be noted that some cancer cells can survive carbon ion radiotherapy and develop improved DSB repair efficacy. Recent pre-clinical studies indicate that the efficacy of radiation therapy can be increased by concomitant treatment with drugs that inhibit DSB repair^23^,^24^. Combining carbon ion radiotherapy with these drugs should be examined in future to see whether they increase anti-tumor efficacy.

The present study has several limitations: (i) we examined only one patient treated with carbon ion radiotherapy due to the limited number of patients who had tumors with accessible biopsy sites and who were not receiving concurrent chemotherapy, which could affect the formation of DSBs; (ii) we did not assess SSBs and base damage, both of which are likely to have an effect on cell killing by high LET irradiation; and (iii) we did not show a direct cause-effect relationship between LET and 53BP1 foci size (this is technically difficult to prove in clinical specimens receiving carbon ion radiotherapy). Comprehensive analysis of DSBs, SSBs, and base damage induced by clinical carbon ion radiotherapy should be performed in a larger cohort to better understand the mechanism underlying the greater anti-tumor effects of carbon ion radiotherapy compared with X-ray radiotherapy.

In summary, this is the first study to visualize complex DSBs in a human tumor treated with carbon ion radiotherapy in a clinical setting. Further in-depth research focused on the DSB complexity is warranted to establish clinical proof of concept that carbon ion radiotherapy has greater anti-tumor effects than X-ray radiotherapy.

## Methods

### Study design

Tumor biopsy specimens were taken from patients with uterine cervical cancer after the first treatment with carbon ion or X-ray irradiation and subjected to immunofluorescence analysis for 53BP1 foci, which are specific markers for DSBs^25^. Foci were visualized using advanced high-resolution microscopy with deconvolution. The size of the 53BP1 foci was measured, and the LET distribution in carbon ions at the center of SOBP and in X-rays was calculated using a physical simulation technique.

The study was approved by the institutional review board of Gunma University Hospital, and carried out in accordance with approved guidelines. Informed consent was obtained from all patients.

### Patients, treatments, and sample collection

Two Japanese patients with newly-diagnosed, locally advanced treatment-naïve uterine cervical cancer who received radical radiotherapy at Gunma University in 2015 were enrolled in the study. One patient was treated with carbon ion radiotherapy. The other patient was treated with X-ray radiotherapy (the detailed therapeutic regimens are described in the Supplementary Methods). Thirty minutes after the first exposure to carbon ions or X-rays, punch biopsies were obtained from the center of the tumor at the uterine cervix (at least 95% of the prescribed dose was delivered to the site of the tumor biopsy; see Supplementary Fig. 8).

### Immunofluorescence staining of biopsy samples

After biopsy, tumor tissues were kept in ice-cold PBS during transport from the hospital to the laboratory. Within 10 min of biopsy, the tumor was minced using a surgical knife and samples incubated in 0.25% trypsin/1 mM EDTA solution at 37 °C for 5 min. The samples were then passed through a 70 micron nylon mesh cell strainer (Falcon) in 5 mL of PBS. Strained cells were collected by centrifugation at 1500 rpm for 5 min and suspended in 0.4 mL of PBS. Samples of cell suspension (0.1 mL) were spread thinly across slide glasses using a Cytospin^TM^ 4 Cytocentrifuge (Thermo Scientific). The slide glasses were then incubated (two rounds) with CSK-R buffer (10 mM PIPES (pH 7.0), 100 mM NaCl, 300 mM Sucrose, 3 mM MgCl_2_, 0.7% TritonX-100, and 0.3 mg/mL RNaseA) for 3 min at room temperature. The cells were then fixed with 3% PFA/2% sucrose for 10 min. Fixed cells were incubated with a 53BP1 antibody (1:1000; Bethyl Laboratories), followed by an Alexa 555-conjugated secondary antibody (1:500; Cell Signaling Technology) and 4′,6-Diamidino-2-Phenylindole, Dihydrochloride (DAPI). Coverslips were mounted in Vectashield (Vector Laboratories).

### Analysis of 53BP1 foci

Microscopic images were obtained under an Applied Precision DeltaVision OMX microscope (GE) using the settings for conventional image capture by a 60× objective lens. A series of 16 images were acquired along the z-axis (0.25 μm intervals over 4 μm) and stacked into a single-layer image with deconvolution. The deconvolved z-stack images were used to measure 53BP1 foci. The maximal width of the 53BP1 foci was measured in 2D using NIH ImageJ 1.48 v. For this study, foci were defined as “individual” if they were >1 μm distant from adjacent foci. A 3D polygon image of the 53BP1 foci was created using Imaris 8.0.1 (Zeiss).

### Physical simulation of LET distribution

LET distribution in carbon ions at the center of the SOBP was calculated using the particle therapy system simulation framework, PTSim^26^, which is a software application within the GEANT4 simulation toolkit^27^,^28^. The standard physics model was applied to the electromagnetic process. The quantum molecular dynamics model, rather than the binary cascade model, was used for inelastic nuclear reactions. For simplicity, only two materials (a scatterer made of lead [6.3 mm thick] and a water phantom [100 × 100 × 500 mm^3^]) were defined. Carbon ions (380 MeV/n) were generated and passed through a scatterer to transfer their energy to the water phantom. The depth dose distribution, i.e., the Bragg curve, and the LET distribution at each depth were calculated in 0.1 mm increments. The depth dose distribution of the SOBP at depth 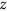, 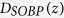, was then calculated by adding the shifted Bragg curves multiplied by weighting factors as follows: 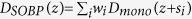, where 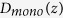 represents the simulated dose of the Bragg curve at depth 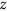, and 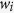 and 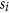 represent the weight and the amount of shift for the *i*-th mono-energetic beam component, respectively. The values of 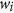 and 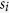 used in this study were the same as those used to design the ridge filter for 110 mm SOBP used to treat the patient. Supplementary Figure 7 shows the simulated physical dose distribution, with schematic Bragg curves.

The LET distribution at the center of the SOBP can be similarly calculated as the sum of weighted LET distributions by a mono-energetic beam as follows: 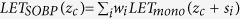, where 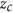, 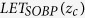, and 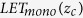 represent the depth at the center of the SOBP, LET distribution by the SOBP, and LET distribution by a mono-energetic beam, respectively. The LET distribution of X-rays (10 MV) at a treatment iso-center depth of 96 mm was also simulated using the same processes.

### Statistical analysis

The significance of the differences in the width of 53BP1 foci after carbon ion and X-ray radiotherapy was examined using the Mann–Whitney U test and SigmaPlot 13 software (Hulinks).

## Additional Information

**How to cite this article**: Oike, T. *et al.* Visualization of complex DNA double-strand breaks in a tumor treated with carbon ion radiotherapy. *Sci. Rep.* **6**, 22275; doi: 10.1038/srep22275 (2016).

## Supplementary Material

Supplementary Information

Supplementary Movie S1

Supplementary Movie S2
