# Supplementary material for: Visualization of complex DNA double-strand breaks in a tumor treated with carbon ion radiotherapy
Source: Sci Rep. 2016 Mar 1;6:22275. doi: 10.1038/srep22275 (PMC4772097; doi:10.1038/srep22275)
Supplement: Supplementary Information [file srep22275-s1.pdf]

Supplementary information

Title:

Visualization of complex DNA double-strand breaks in tumor treated by carbon ion radiotherapy

Authors: Takahiro Oike[1,2]#, Atsuko Niimi[3]#, Noriyuki Okonogi[1], Kazutoshi Murata[1,2], Akihiko Matsumura[2], Shin-Ei Noda[1,2], Daijiro Kobayashi[1], Mototaro Iwanaga[1], Keisuke Tsuchida[1], Tatsuaki Kanai[2], Tatsuya Ohno[2], Atsushi Shibata[4]*, Takashi Nakano[1,2]*.

#These authors contributed to this work equally.

Affiliations: [1]Department of Radiation Oncology, Gunma University Graduate School of Medicine, 3-39-22, Showa-machi, Maebashi, Gunma, 371-8511, Japan; [2]Gunma University Heavy Ion Medical Center, 3-39-22, Showa-machi, Maebashi, Gunma, 371-8511, Japan; [3]Research Program for Heavy Ion Therapy, Division of Integrated Oncology Research, Gunma University Initiative for Advanced Research, 3-39-22, Showa-machi, Maebashi, Gunma, 371-8511, Japan; [4]Advanced Scientific Research Leaders Development Unit, Gunma University, 3-39-22, Showa-machi, Maebashi, Gunma, 371-8511, Japan.

***Corresponding author:**

Atsushi Shibata, PhD

Tenure Track Assistant Professor

Advanced Scientific Research Leaders Development Unit, Gunma University

3-39-22, Showa-machi, Maebashi, Gunma 371-8511, Japan.

Tel.: +81-027-220-7977; Fax: +81-027-220-7909; E-mail: shibata.at@gunma-u.ac.jp

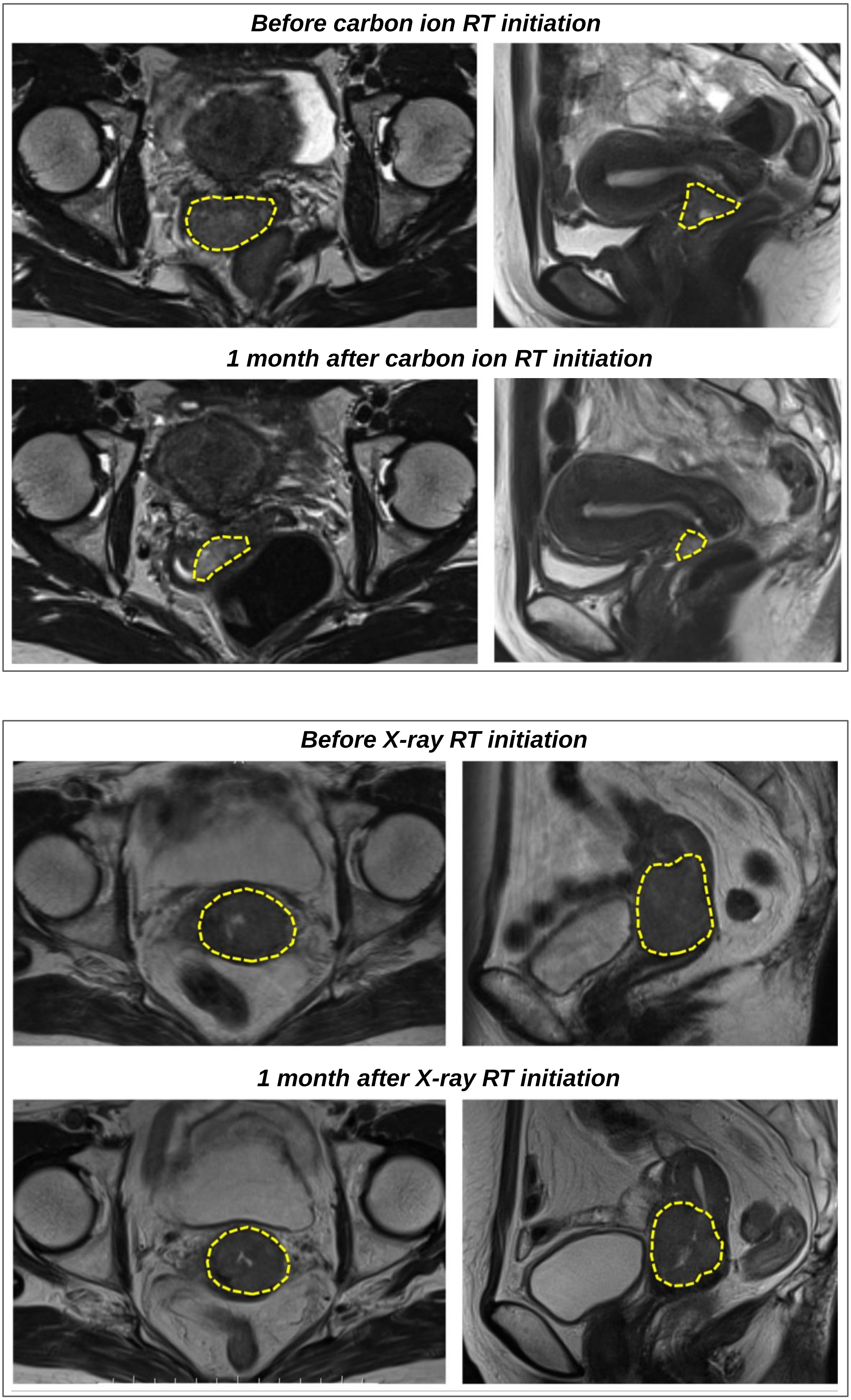

Supplementary Figure 1. Magnetic resonance imaging (T2WI) of treated tumors. Tumors are delineated by yellow dotted lines. RT, radiotherapy.

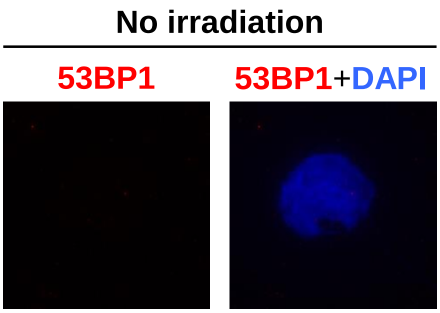

Supplementary Figure 2. Representative microscopic image showing the absence of 53BP1 foci in a non-irradiated specimen. Tumor tissue was taken by punch biopsy at the time of diagnosis (before initiation of radiotherapy) from the patient received carbon ion radiotherapy, and stained with 53BP1 and DAPI.

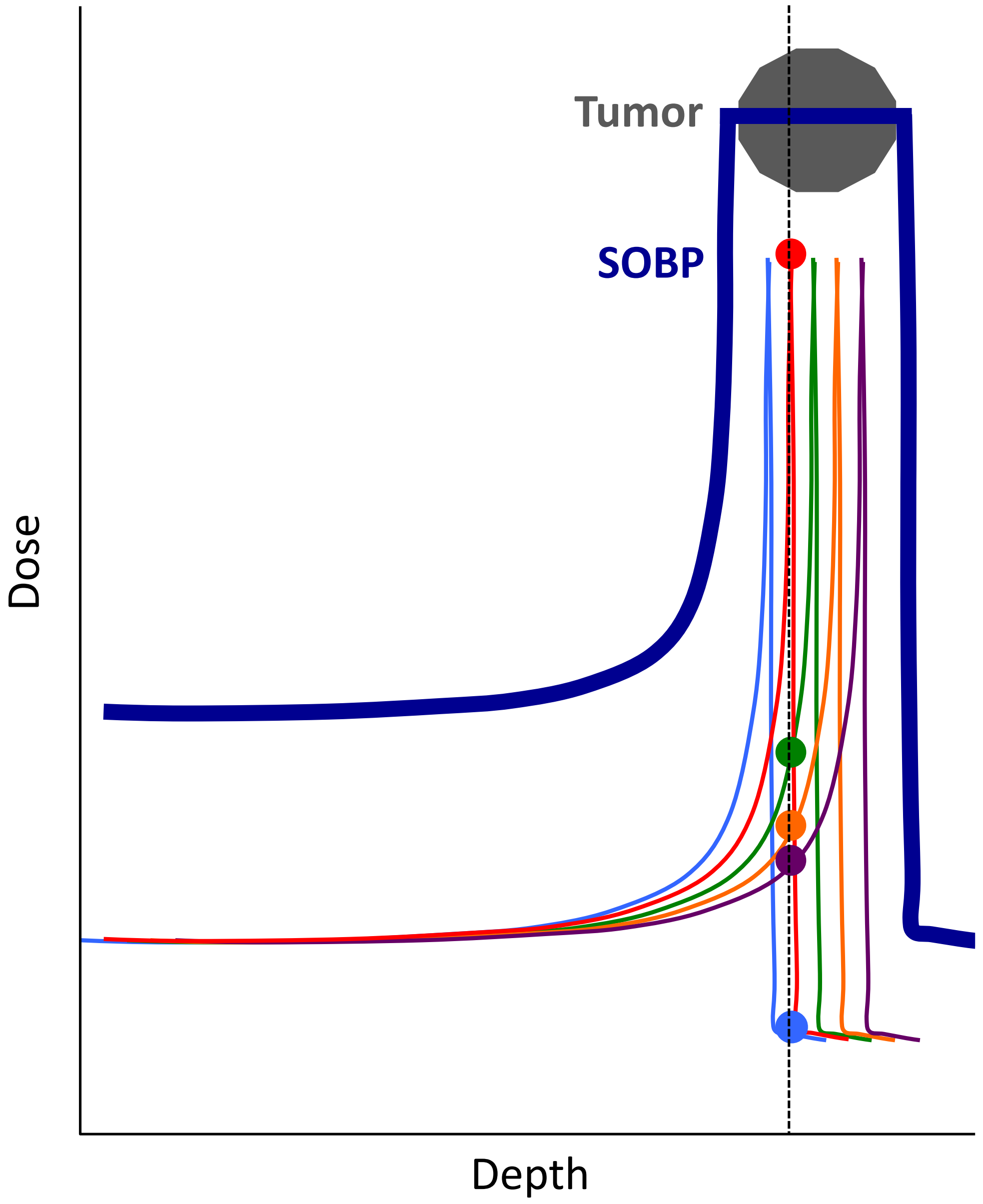

Supplementary Figure 3. Schematic presentation demonstrating SOBP adjusted to tumor size. SOBP carbon ion beams are generated by integrating mono-energetic carbon ion beams that shift gradually in depth. At a given point (indicated by a black dashed line), cancer cells receive mixture of beams (indicated by colored dots) with varying LET. See also Supplementary Fig 5 for the depth-LET association in a Bragg curve of mono-energetic beam.

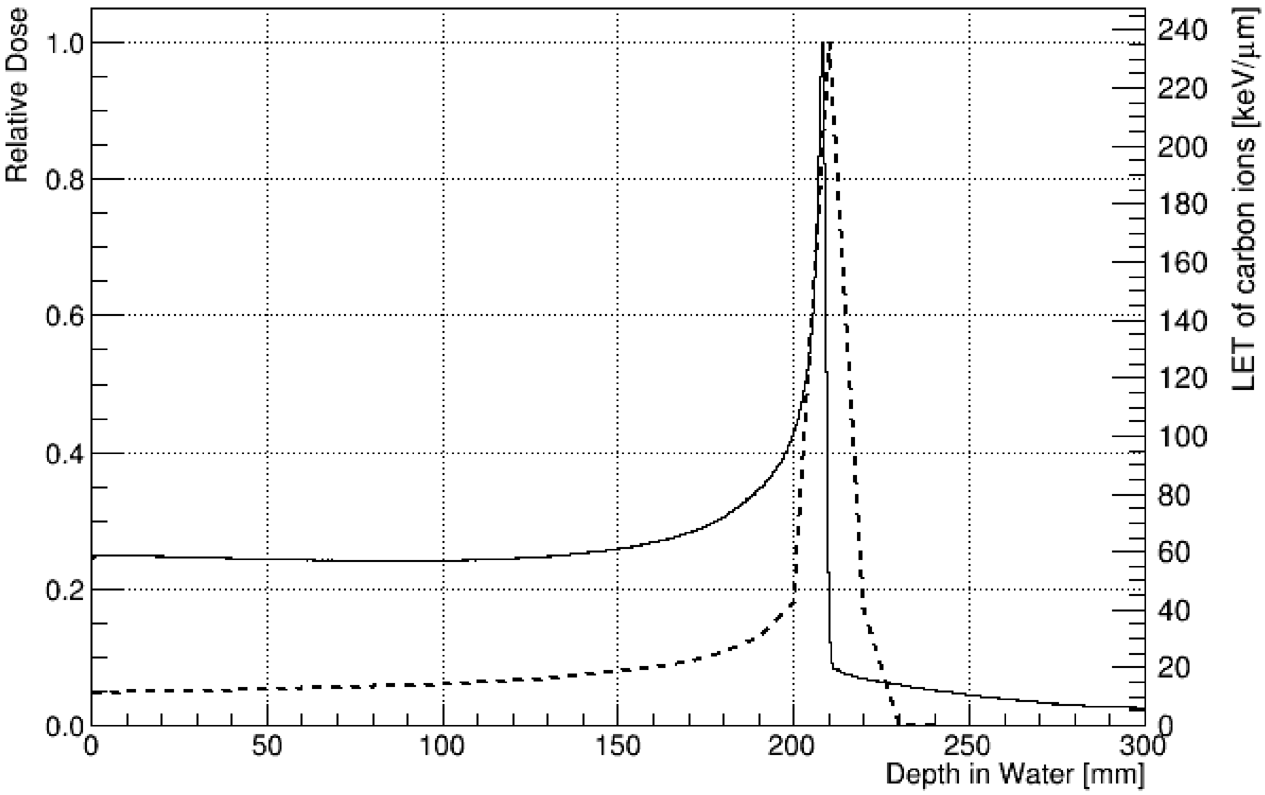

Supplementary Figure 4. Depth-dose and depth-LET distribution for a 380 MeV/n mono-energetic carbon ion beam in water. Solid line and dashed line represents the physical dose and LET, respectively, demonstrating the sharp peak in LET distribution that is corresponding to the sharp peak in the dose distribution (Bragg peak).

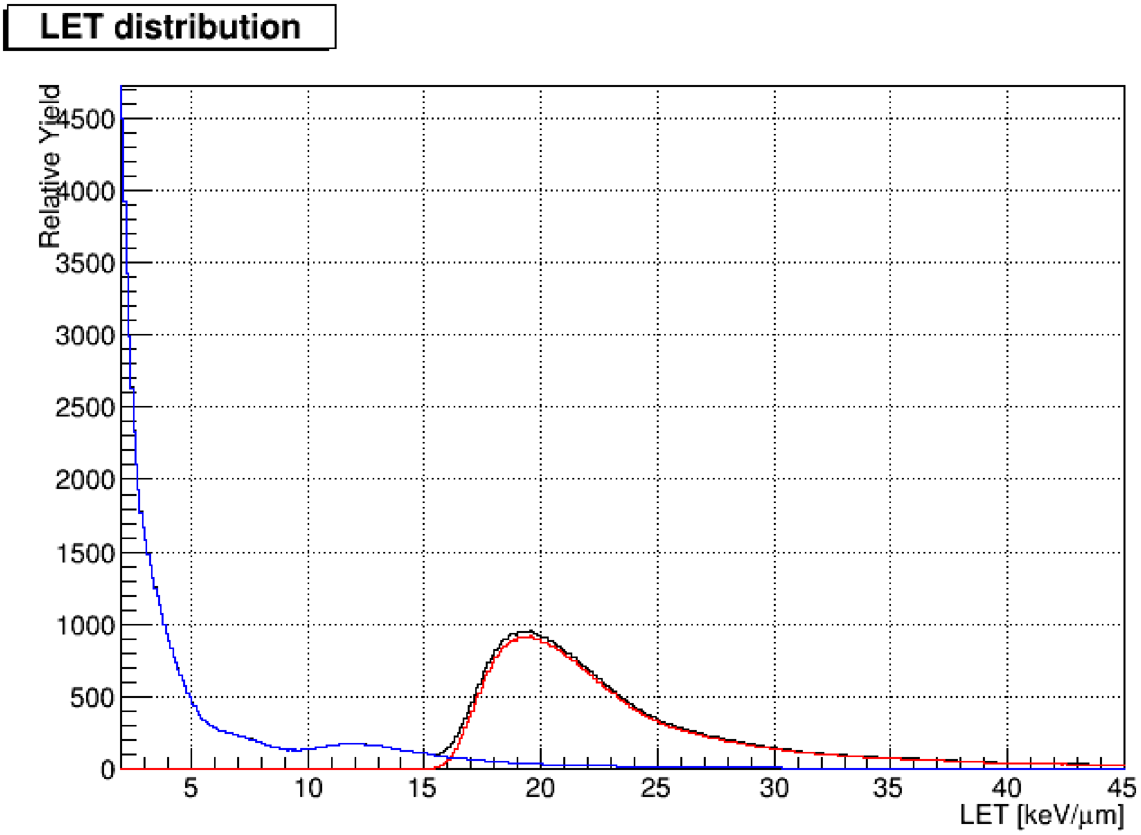

Supplementary Figure 5. LET distribution of carbon ion beams at the center of SOBP calculated by physical simulation. Red line and blue line indicate the distribution of carbon ions and secondary charged particle, respectively. Details of physical simulation are described in Methods section.

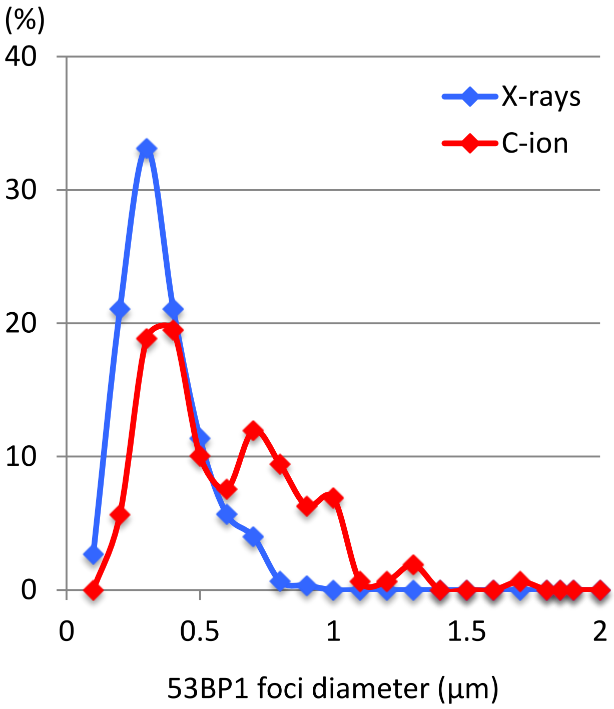

Supplementary Figure 6. The distribution of 53BP1 foci number according to the foci width in carbon ion- and X-ray-irradiated specimens. The graphs in Figure 3B and 3D are merged, indicating that the lower peak in the carbon ion-irradiated specimen is well consistent with the peak in the X-ray-irradiated specimen.

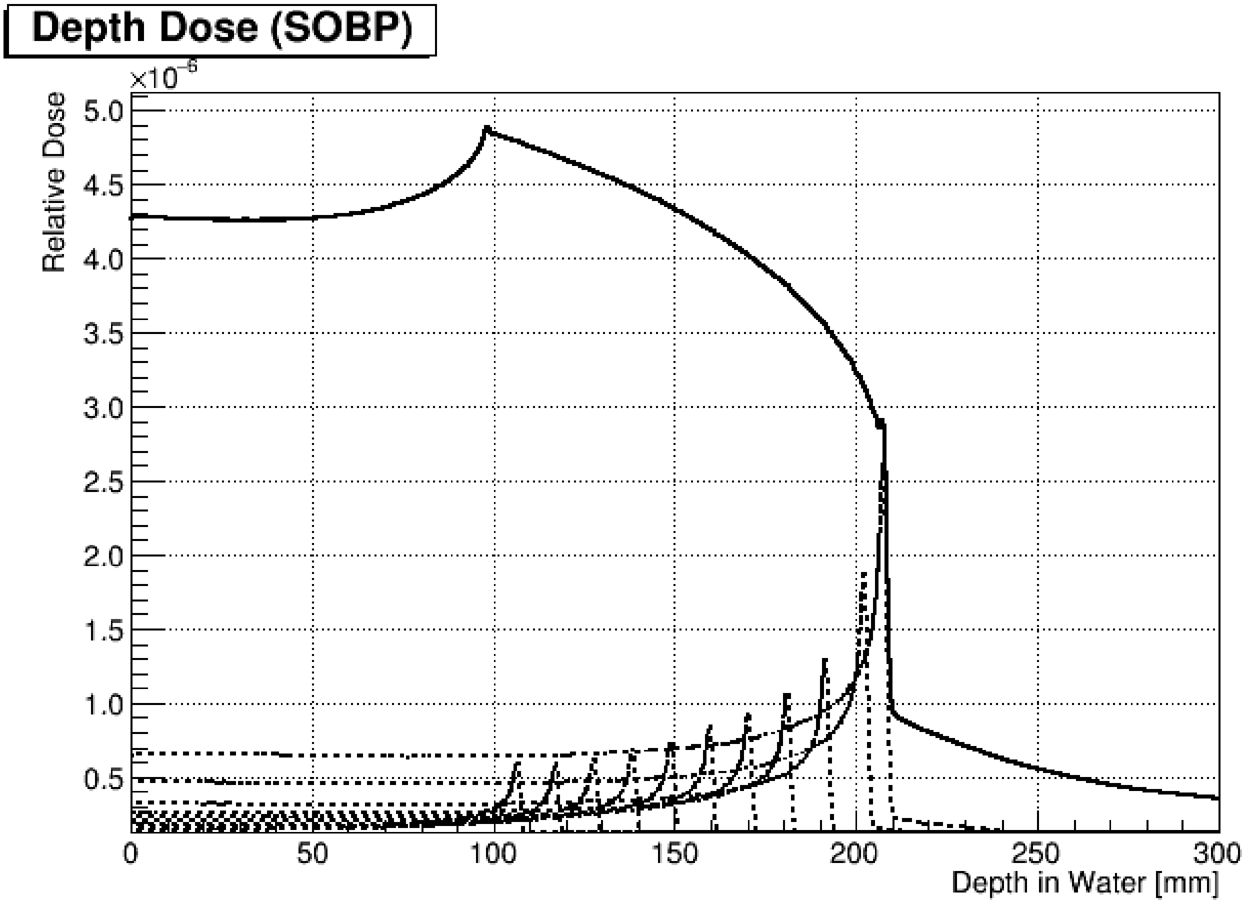

Supplementary Figure 7. Simulated depth dose distribution for 380 MeV/n carbon ions in water. Solid line and dashed lines represent the physical dose for the SOBP width of 110 mm and schematic Bragg curves for mono-energetic beam components, respectively.

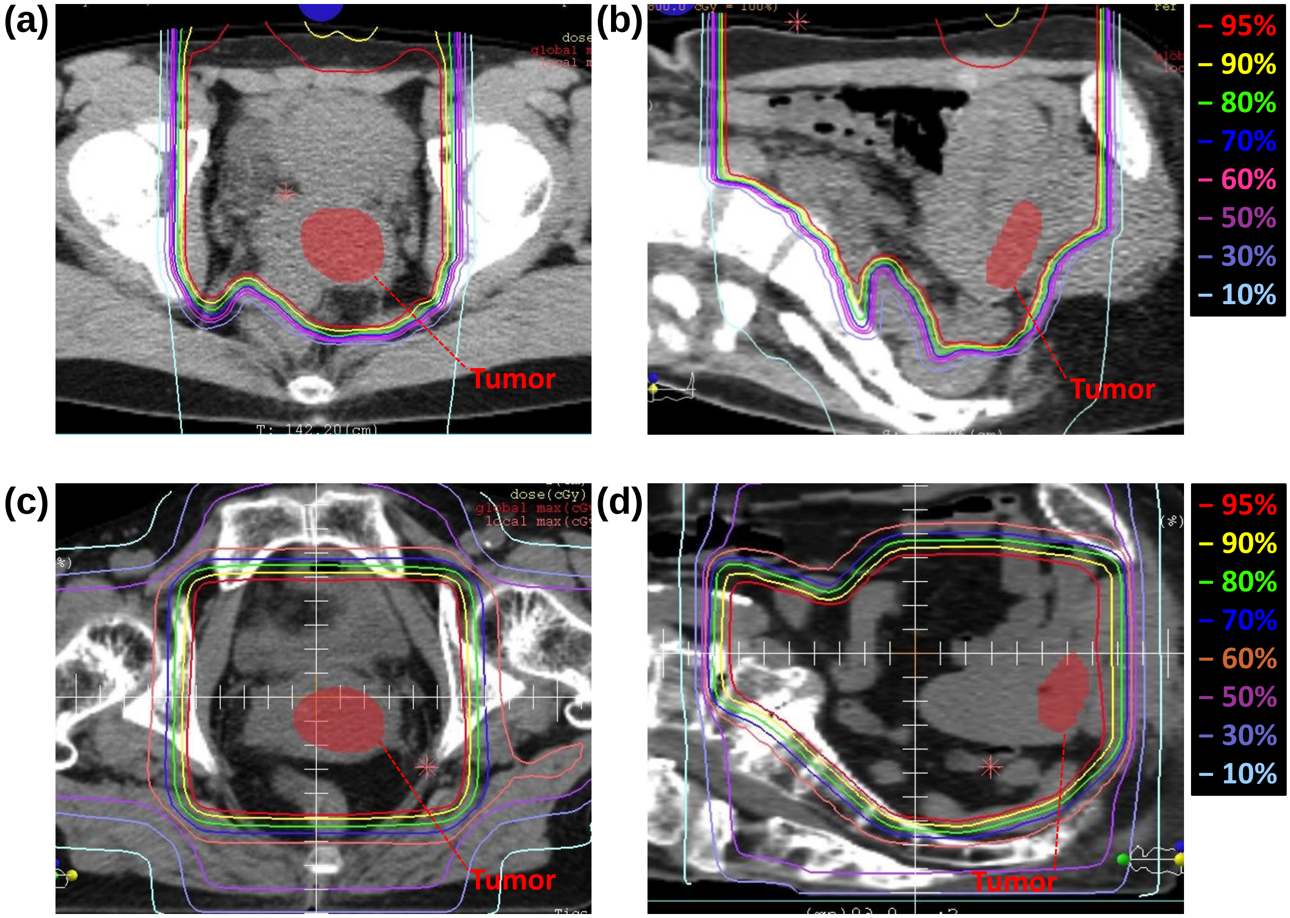

Supplementary Figure 8. Representative computed tomography images showing dose distributions of carbon-ion and X-ray radiotherapy at Day 1. Colored lines show percentage prescribed dose, indicating that tumors are covered by at least 95% of the prescribed doses. (a, b) Carbon ion radiotherapy. A transverse plane (a); a saggital plane (b). Beams are delivered from anterior direction. (c, d) X-ray radiotherapy. A transverse plane (c); a saggital plane (d). X-rays are delivered from anterior, posterior and bilateral directions.

Supplementary Table 1. Patient Characteristics

	Patient #1	Patient #2
Modality	Carbon ions	X-rays
Age	43	76
Sex	Female	Female
Histology	Adenocarcinoma	Squamous cell carcinoma
FIGO stage	IIB	IIB
Tumor size (*a* x *b* x *c* mm)		
Before RT initiation	*34* x *26* x *23*	*52* x *35* x *41*
1M after RT initiation	*26* x *20* x *16*	*45* x *33* x *39*
Change in tumor volume (%)	-59	-22

Abbreviations: FIGO, International Federation of Gynecology and Obstetrics; *a* -*c* , tumor diameter in three mutually orthogonal dimensions in magnetic resonanse imaging (T2 weighted imaging); RT, radiotherapy; M, month. Tumor volume was calculated by the formula: tumor volume = *abc* /2.

Supplementary Discussion

Cluster DSB foci were analyzed by immunofluorescence staining of 53BP1 using fresh tumor specimens

To visualize DSBs in tumor biopsy specimens, we performed immunofluorescence staining for 53BP1 rather than for γH2AX, which is a well-known DSB marker. This is because γH2AX generates a high background signal in the specimens. In addition, the edges of γH2AX foci within the specimens were obscure. These factors made it difficult to perform precise foci measurements. Similar to γH2AX foci, 53BP1 accumulates and forms foci at sites of DSBs[1]. *In vitro* studies show that the number of 53BP1 foci is closely correlated with the number of DSBs after X-ray irradiation[2,3]. Furthermore, the kinetics of 53BP1 appearance and disappearance are very similar to those of γH2AX. In fact, 53BP1 was recently used as a specific DSB marker. A recent study also showed that kinetics of formation and decay of 53BP1 foci is associated with radiosensitivity, suggesting that 53BP1 foci can be a good marker for DSBs after ionizing irradiation[4]. Thus, we used 53BP1 for DSB analysis. Some studies used immunohistochemistry or immunofluorescence staining to show DSB formation in fresh-frozen clinical samples[5]. However, freezing biopsy samples obscures the edges of the foci signals (data not shown). Therefore, we considered fresh-frozen samples unsuitable for high-resolution analysis and so performed immunofluorescence staining immediately after biopsy.

Tumor biopsy was performed 30 min after the first irradiation

In a previous study, peak γH2AX foci formation was observed at 15–30 min after X-ray irradiation[3]. Therefore, we obtained tumor biopsies 30 min after irradiation. Carbon ion and X-ray radiotherapy were performed daily; thus biopsy samples taken after irradiation on Day 2 or later will have contained DSBs resulting from different irradiations. On the other hand, the size of the clustered DSB foci decreases with time due to DSB repair[6]. Thus, to examine the size distribution of DSBs induced by a single fraction of radiotherapy, we performed tumor biopsy

after Day 1 of irradiation.

Supplementary Methods

Radiotherapy regimens

Carbon ion radiotherapy comprised whole pelvic irradiation (12.0 Gy in 12 fractions), followed by a radiation boost to the tumor (4.8 Gy in four fractions), on a 4 days-per-week schedule. Whole pelvic irradiation was performed using anterior and posterior ports, and boost irradiation was performed using anterior and lateral ports. Note that the doses for carbon ion radiotherapy were based on the relative biological effectiveness of carbon ion beams compared with X-rays; this value is 3[7]. Carbon ion radiotherapy was followed by three sessions of computed tomography-guided high-dose-rate intracavitary brachytherapy (HDR-ICBT) using an iridium-192 source over the course of 2 weeks; during each session, at least 5.4 Gy was delivered to the tumor and the uterine cervix.

X-ray radiotherapy comprised whole pelvic irradiation (30 Gy in 15 fractions) and pelvic irradiation with central shielding (20 Gy in ten fractions), on a 5 days-per-week schedule; the former was delivered using the four-field box technique, and the latter was delivered using a parallel-opposed two-field technique. From the fourth week, patients received four sessions of HDR-ICBT (one session per week); during each session, at least 5.4 Gy was delivered to the tumor and the uterine cervix.

Supplementary References

1. Schultz. L. B., *et al*. p53 binding protein 1 (53BP1) is an early participant in the cellular response to DNA double-strand breaks. *J Cell Biol* **151**, 1381–1390 (2000)
2. Löbrich. M., *et al*. gammaH2AX foci analysis for monitoring DNA double-strand break repair: strengths, limitations and optimization. *Cell Cycle* **9**, 662–669 (2010)
3. Stiff. T., *et al*. ATM and DNA-PK function redundantly to phosphorylate H2AX after exposure to ionizing radiation. *Cancer Res* **64**, 2390–2396 (2004)
4. Markova. E., *et al*. 53BP1 foci as a marker of tumor cell radiosensitivity. *Neoplasma* **62**, 770–776 (2015)
5. Bonner. W. M., *et al*. GammaH2AX and cancer. *Nat Rev Cancer* **8**, 957–967 (2008)
6. Nakajima. N. I., *et al*. Pre-exposure to ionizing radiation stimulates DNA double strand break end resection, promoting the use of homologous recombination repair. *PLoS One* **10**, e0122582 (2015)
7. Kanai. T., *et al*. Biophysical characteristics of HIMAC clinical irradiation system for heavy-ion radiation therapy. *Int J Radiat Oncol Biol Phys* **44**, 201-210 (1999)

Legend to the Supplementary Movie 1 and 2

Video showing 3D rendering of the nucleus in a carbon ion-irradiated cell (Supplementary Movie 1) and the nucleus in an X-ray-irradiated cell (Supplementary Movie 2). 3D rendering of a single nucleus was performed using Imaris 8.0.1 (Zeiss). Cells were stained with 53BP1 (orange) and DAPI (blue). The polygon image of 53BP1 was generated using Imaris 8.0.1.
